# More functions of torpor and their roles in a changing world

**DOI:** 10.1007/s00360-017-1100-y

**Published:** 2017-04-21

**Authors:** Julia Nowack, Clare Stawski, Fritz Geiser

**Affiliations:** 1 Centre for Behavioural and Physiological Ecology, Zoology, University of New England, Armidale, NSW 2351 Australia; 2 Department of Integrative Biology and Evolution, University of Veterinary Medicine, Vienna, Savoyenstraße 1, Vienna, 1160 Austria

**Keywords:** Colonization, Evolution, Fires, Heat, Heterothermy, Storm

## Abstract

Increased winter survival by reducing energy expenditure in adult animals is often viewed as the primary function of torpor. However, torpor has many other functions that ultimately increase the survival of heterothermic mammals and birds. In this review, we summarize new findings revealing that animals use torpor to cope with the conditions during and after natural disasters, including fires, storms, and heat waves. Furthermore, we suggest that torpor, which also prolongs longevity and was likely crucial for survival of mammals during the time of the dinosaur extinctions, will be advantageous in a changing world. Climate change is assumed to lead to an increase in the occurrence and intensity of climatic disasters, such as those listed above and also abnormal floods, droughts, and extreme temperatures. The opportunistic use of torpor, found in many heterothermic species, will likely enhance survival of these challenges, because these species can reduce energy and foraging requirements. However, many strictly seasonal hibernators will likely face the negative consequences of the predicted increase in temperature, such as range contraction. Overall, available data suggest that opportunistic heterotherms with their flexible energy requirements have an adaptive advantage over homeotherms in response to unpredictable conditions.

## Introduction

The reduction of energy expenditure, achieved by a controlled reduction of metabolic rate and body temperature (*T*_b_), during cold periods that often coincide with low food availability is frequently viewed as the ultimate benefit of torpor (Lyman et al. 1982). Indeed, many species use torpor mainly in response to such seasonal bottlenecks (Brigham et al. 2000). However, in the past decade, it has become evident that torpor has more functions than just energy conservation for adult mammals and birds during winter (reviewed in Geiser and Brigham 2012). For example, torpor can enhance fat storage during migration, or permit birds to remain resident during winter and maintain and defend their territories year round (Geiser and Brigham 2012), to name only a few additional functions of torpor (for more functions see Table 1). Since the original suggestion of “other functions” of torpor by Geiser and Brigham (2012), the list has continued to grow (Table 1, 2). Recently identified functions include coping with extreme heat (Bondarenco et al. 2014), facilitating (island) colonization (Nowack and Dausmann 2015), enabling mammalian survival at the Cretaceous-Palaeogene (K-Pg) boundary (Lovegrove et al. 2014a) and perhaps a prolonged lifespan (Lyman et al. 1981; Turbill et al. 2011). Importantly, one of the major recent findings adding to the varied benefits of torpor is that animals can use torpor to cope with detrimental conditions during and after natural disasters, such as fires and storms (e.g., Nowack et al. 2015; Stawski et al. 2015a). It has already been noted previously that the reduction in metabolic rate during torpor lowers the need of food intake, likely enabling animals to decrease the time spent actively foraging (Geiser and Brigham 2012). Heterothermic species (those that can employ torpor or those that increase/decrease their *T*_b_ only slightly above/below normothermic levels) are less often subjects of extinction than homeothermic species (that maintain a stable high *T*_b_), suggesting that the reduction of energy expenditure and its additional benefits substantially increase the chance of survival of individuals (Geiser and Turbill 2009; Hanna and Cardillo 2014). Hibernating mammals can survive for months without food and water intake in their hibernacula, where they are somewhat independent of the conditions on the earth’s surface. Edible dormice (*Glis glis*) are an extreme example as they hibernate for 11 months in non-reproductive years, presumably to avoid predation during years when conditions are not favorable for reproduction (Bieber and Ruf 2009; Hoelzl et al. 2015). Similarly, eastern pygmy-possums (*Cercartetus nanus*) can hibernate for up to an entire year without feeding (Geiser 2007). We hypothesize that heterothermic mammals may have an advantage over homeothermic species as they can remain hidden and protected during short or long periods of torpor. Moreover, it is likely that opportunistic torpor will be advantageous in a world subjected to climate change. In this paper, we review the functions of torpor that have been reported since the original review by Geiser and Brigham (2012) and discuss whether and how some of these functions may help animals to deal with the predicted environmental changes.

**Table 1:** List of the other functions of torpor that were already listed in Geiser and Brigham 2012 including additional supporting references that were published after 2012

Function/challenge	Benefits of torpor	References
Migration	Increases fat storage prior to migration	Carpenter and Hixon (1988); Hiebert (1993); Körtner et al. (2000)
	Allows maintenance of year round home range	
Reproduction	Enhances sperm storage	Morton (1978); Racey and Swift (1981); Audet and Fenton (1988); Birkhead and Møller (1993); Geiser and Masters (1994); Grinevitch et al. (1995); Willis et al. (2006); Körtner et al. (2008); Wang et al. (2008); Morrow and Nicol (2009); Canale et al. (2012); McAllan and Geiser (2014); Stawski and Rojas (2016)
	Delays parturition	
	Enhances body condition prior to lactation	
	Water/energy conservation	
	Permits reproduction on limited resources	
Development	Permits and enhances development and growth on limited food resources	Nagel (1977); Geiser et al. (2006); Giroud et al. (2012); Giroud et al. (2014)
Water conservation	Reduces water loss	MacMillen (1965); Buffenstein and Jarvis (1985); Withers et al. (1990); Hosken and Withers (1997); Cooper et al. (2005); Schmid and Speakman (2009)
Drought	Survival of long periods of reduced water availability	Doucette et al. (2012)
Parasites/disease	Removal of parasites	Callait and Gauthier (2000); Fietz et al. (2014) Chute (1964); Lourenço and Palmeirim (2008); Fietz et al. (2016)
	Removal of spirochaetes	
Inter-specific competition	Allows co-existence of competing species during periods of resource limitation and high energetic requirements	Levy et al. (2011)
Extinction	Allows long-term survival even under adverse environmental conditions	Bieber and Ruf (2009); Geiser and Turbill (2009); Liow et al. (2009); Stawski and Geiser (2010); Turbill et al. (2011); Hanna and Cardillo (2014); Hoelzl et al. (2015)
	Predator avoidance

**Table 2:** List of the newly identified other functions of torpor relevant to climate change

Function/challenge	Benefits of torpor	References
Fires	Allows individuals to remain inactive during a fire	Stawski et al. (2015a); Nowack et al. (2016a); Matthews et al. (2017)
	Allows survival on limited food resources	
	Predator avoidance	
Storms	Allows individuals to remain inactive during a storm	Willis et al. (2006); Nowack et al. (2015)
Colonization	Allows survival during raft/journey through harsh environments	Nowack and Dausmann (2015)
	Facilitates establishment of founder population	
	Facilitates survival in new climate	
Extreme heat	Reduces evaporative water loss	Schmidt-Nielsen et al. (1956); Song et al. (1997); Grimpo et al. (2013); Bondarenco et al. (2014)
	Delays/shortens the time period in which cooling is required	
Evolution of mammals	Allowed survival during global wildfires/nuclear winter following the meteoroid impact	Lovegrove et al. (2014a); Nowack et al. (2016a)
Longevity	Increases survival probability	Lyman et al. (1981); Carey et al. (2003); Turbill et al. (2011); Turbill et al. (2012); Blanco and Godfrey (2013); Blanco and Zehr (2015)
	Allows spreading of reproduction over several years	
	Reduces reactive oxygen (ROS)	
	Delays physiological aging	

## Heterothermy, extreme heat, and water conservation

Both severe cold and extreme heat result in thermal stress for organisms. From a short-term survival point of view, extreme heat is even more dangerous than cold, especially in small mammals (e.g., Welbergen et al. 2008). Prolonged exposure to ambient temperatures (*T*_a_) above the thermo-neutral zone results in high rates of evaporative water loss during the daytime and consequently water stress, rather than or in addition to energy shortage, may be a major cue for torpor expression in desert species (Geiser 2004). While heterothermic species can decrease their *T*_b_ and undergo torpor, they often can also thermoconform at high *T*_a_s and increase *T*_b_ to hyperthermic values. Over 60 years ago, Schmidt-Nielsen et al. (1956) discovered that camels (*Camelus dromedarius*) exposed to extreme heat in a desert, increased daily amplitudes in *T*_b_ from ~2 to >6 °C when water was restricted. Importantly, the increase in daily *T*_b_ amplitude was achieved by both a predictive reduction in *T*_b_ in the cooler morning as well as a further increase in *T*_b_ during the warmer part of the day. Small desert bats have an even greater heat tolerance than camels. While *T*_b_ of camels increased to above 41 °C, skin temperature (*T*_skin_) in *Mormopterus petersi* increased to 45.8 °C during a heat wave. Therefore, these bats can tolerate the most extreme *T*_b_ range known for mammals, ranging from *T*_skin_ of 3.3 °C during torpor in winter to 45.8 °C during extreme heat (Bondarenco et al. 2014). Similarly, torpor use even within the thermo-neutral zone has been reported in eastern pygmy-possums (*Cercartetus nanus*) and golden spiny mice (*Acomys russatus*) at *T*_a_s as high as 30°–35 °C, whereby metabolic depression was associated with only small decreases in *T*_b_ (Song et al. 1997; Grimpo et al. 2013). The advantage of thermoconforming at high *T*_a_ is the concurrent reduction in metabolic rate and the associated decrease of evaporative water loss (see Geiser and Brigham 2012). Camels, for example, maintain a stable *T*_b_ and use evaporative cooling when water is available, but use “adaptive heterothermy” (in birds termed ‘facultative hyperthermia’; see Tieleman and Williams 1999), i.e., increased daily amplitudes in *T*_b_, when water stressed, thereby reducing water requirements enormously.

Torpor use to save water might be more essential for survival than the related energy savings (e.g., Cooper et al. 2005; Schmid and Speakman 2009; Dausmann 2014; Lovegrove et al. 2014b). Evaporative water loss can be decreased to below measurable levels during torpor (Withers et al. 1990) and the reduction in energy expenditure at high *T*_a_ does not only decrease evaporative water loss by reduced respiration, but also lowers the endogenous thermal load which further reduces the need for evaporative cooling (Cooper et al. 2005). Bats that undergo torpor during hot days in the desert displayed longer torpor bouts at a given *T*_a_ than bats from temperate and subtropical habitats (Bondarenco et al. 2013). A low *T*_b_ during torpor will delay the time until a critically high *T*_b_ is reached and shortens the time required to use evaporative cooling during the hot part of the day (Bondarenco et al. 2013).

## Torpor use and fires

Heat is not only a challenge for a mammal’s water budget, but also increases the frequency of wildfires. Regular wildfires during summer are a threat for many mammals and are especially dangerous for small-bodied terrestrial species that cannot easily flee. Nevertheless, post-fire surveys suggest that some small-to-medium sized mammals survive fires by hiding in rock cavities or underground borrows (Matthews et al. 2017). For example, short-beaked echidnas (*Tachyglossus aculeatus*) became inactive and entered multiday torpor during a fuel-reduction burn in their natural habitat (Nowack et al. 2016a). Although the echidnas mostly used tree logs that were not necessarily safe during a fire, the use of multiday torpor allowed them to remain inactive, reducing the risk of getting trapped by the fire while foraging. After the fire, echidnas in burnt areas decreased their activity and remained inactive for 2–5 days. In contrast to homeothermic animals, which have to resume foraging even under adverse conditions because of their constant high energy demands, heterothermic animals can compensate for lost foraging opportunities by remaining inactive and reducing energy demands through the use of torpor.

Fires not only pose an acute risk to animals, but also destroy food resources and leave the surviving animals in a fire-scorched landscape. How an individual will cope with conditions after a fire depends on the individuals’ behaviour and physiology. Studies on the abundance of mammals after fires indicate that heterothermic species, such as yellow-footed antechinus (*Antechinus flavipes*), have higher survival rates than homeothermic species, such as bush rats (*Rattus fuscipes*) (Thompson et al. 1989; Recher et al. 2009). Antechinus are known to use daily torpor, whereas available data on bush rats suggest that they are homeothermic (Geiser and Körtner 2010). Post-fire studies on heterothermic species suggest that individuals reduce activity and increase torpor use for weeks after a fire to enhance survival (Stawski et al. 2015a; Nowack et al. 2016a; Matthews et al. 2017). In the short-beaked echidna, increased torpor use after the fire likely compensates for reduced food availability and destroyed nesting sites (Nowack et al. 2016a). Echidnas feed on ants and termites, which are limited after the fire has burned woody debris. However, in addition to reduced food availability, these animals also have to deal with diminished ground cover. Small animals will have less shelter during activity and, therefore, face an additional increased risk of predation (Radford 2012). Consequently, torpor use in the small marsupial brown antechinus (*A stuartii*, ~16–40 g) is assumed to increase survival chances by limiting the time spent foraging while exposed to predators (Stawski et al. 2015a). The fact that neither echidnas, nor antechinus left their fire-destroyed habitats indicates that torpor use after fires allows animals to remain in scorched landscapes and reduces the need to re-populate habitats after a fire (Stawski et al. 2015a; Matthews et al. 2017). A long-term study on a population of brown antechinus (*A. stuartii*) found that the population had returned to normal activity and torpor use within a full year after a prescribed fire (Hume 2015).

Although increasing torpor use after a fire is beneficial for terrestrial mammals, such as the echidna and antechinus, microbats (insect eating bats) appear to respond differently. Torpor use and activity periods were monitored in lesser long-eared bats (*Nyctophilus geoffroyi*) 4 months after a severe wildfire and again 2 years later (Doty et al. 2016). These authors found that shortly after the wildfire, bats expressed shorter torpor bouts and longer normothermic periods in comparison with 2 years after the wildfire (Doty et al. 2016). As aerial insect abundance was significantly greater shortly after the fire and ambient conditions were warmer, it is likely that bats took advantage of the increase in insect numbers and also in the decrease in vegetation, which would have made foraging easier (Doty et al. 2016). As flying mammals, bats also have the advantage of being able to more easily flee a fire and then return to their home ranges once the fire has passed. These studies reveal that different animals respond in various ways to fire, highlighting the importance of obtaining data on diverse species in this context.

## Torpor use and the evolution of mammals

The ability of hibernators to disappear from the surface and hibernate for months in underground burrows has also been suggested to have facilitated the survival of mammals during the meteorite impact at the K-Pg boundary, about 65.5 million years ago (Lovegrove et al. 2014a). This event killed the dinosaurs and many other vertebrate species by causing global wildfires (Morgan et al. 2013). The mammals that survived the meteoroid impact would have had to deal initially with global wildfires, followed by reduced light levels for a year or more, likely leading to a reduction of *T*_a_, both of which would have significantly reduced food availability (Robertson et al. 2004). As we describe above, data on torpor use during and after fires support the hypothesis by Lovegrove et al. (2014a). Torpor use in protected shelters would not only have been beneficial for the survival of the animals during the cold, but also during the blazing fires (Stawski et al. 2015a; Nowack et al. 2016a). In this regard, it is particularly interesting that especially echidnas enter torpor in response to fires and were found as one of the few survivors after a severe wildfire (Nowack et al. 2016a; Matthews et al. 2017). Echidnas have been described as “protoendotherms” and have many ancestral traits, such as a relaxed thermoregulation and the ability to frequently use torpor (Grigg et al. 2004), and these traits likely resemble traits of ancestral monotremes at the time of the K-Pg boundary.

Hibernation in temperate zone animals is usually characterized by torpor bouts lasting up to 5 weeks, interspersed by regular arousal phases in which the animal rewarms to euthermic *T*_b_s for a few hours (Carey et al. 2003). Periodic euthermic phases are energetically costly and in temperate species, the greatest proportion (70–80%) of the energy expended during hibernation is used for these rewarming processes (Wang 1978). In contrast, some tropical species, such as tenrecs or lemurs, can undergo hibernation periods without this characteristic periodic arousal process, but in these species, *T*_b_ closely tracks daily fluctuations in *T*_a_ (Dausmann et al. 2004; Kobbe et al. 2011; Lovegrove et al. 2014a). The original suggestion that torpor use may have facilitated mammalian survival at the K-Pg boundary was based on the observation of long-term hibernation without regular interbout arousals in the common tenrec (*Tenrec ecaudatus*), another “protoendotherm” [termed “basoendotherm” by Lovegrove (2012)] (Lovegrove et al. 2014a). Torpor use with less frequent costly arousals would have led to even greater energy savings and could have enabled ancestral mammals to survive until conditions on the surface had eased (Lovegrove et al. 2014a).

## Heterothermy and storms

Storms are experienced by animals worldwide and, unlike fires, can occur anywhere. However, evidence for torpor use during storms is rare, and to date has only been reported for two species, the hoary bat (*Lasiurus cinereus*) and the marsupial sugar glider (*Petaurus breviceps*) (Willis et al. 2006; Nowack et al. 2015). Pregnant hoary bats used torpor during a snow storm in the Canadian spring, not only reducing the need to forage during adverse conditions, but also delaying parturition until conditions were more favorable (Willis et al. 2006). Individuals stayed inactive for a maximum of 9.1 days and the longest torpor bout recorded lasted 5.6 days. However, torpor use might not have been caused by the storm itself, but rather by the low *T*_a_ of ~5 °C or by a combination of cold exposure and convective heat loss. In contrast, sugar gliders, which rarely used torpor before the storm, used highly synchronized torpor during spring in a warm, subtropical habitat during a cyclone. During this storm, *T*_a_ did not decrease below values observed on other nights, and therefore, torpor use likely occurred in response to high wind speed and rainfall (Nowack et al. 2015).

From the available data, it is not clear whether gliders or bats anticipated the storm, but since some sugar gliders remained completely inactive during the storm night, it appears that they responded proactively to environmental cues, such as changes in barometric pressure, that were preceding the storm. No data exist on perception of barometric pressure changes in sugar gliders. However, studies on hibernating brown bats (*Myotis lucifugus*) and eastern pipistrelles (*Pipistrellus subflavus*) have suggested that bats use changing barometric pressure as a cue for conditions outside their hibernacula. Bats emerge from torpor when falling barometric pressure indicates favorable foraging conditions (Paige 1995; Czenze and Willis 2015).

## Torpor use and colonization

Torpor use during unpredictable situations, such as storms or fires, also support the hypothesis that torpor can be advantageous during over-water colonization events (Nowack and Dausmann 2015). This hypothesis proposes that land masses or tree logs that contain mammals could be washed into the ocean during a storm event (e.g., Simpson 1940). As a consequence, the animals would unexpectedly find themselves without access to food or drinkable fresh water for a long period of time. Opportunistic torpor use would allow animals to cope with these conditions until eventually arriving on a shore. Importantly, torpor use would not only be an advantage during the rafting event (Martin 1972; Kappeler 2000), but also after arrival in the new habitat (Nowack and Dausmann 2015). The arriving animals would face unfamiliar terrain and ambient conditions, including a lack of known food sources. Furthermore, torpor use could facilitate the establishment of a founder population, by enabling a reproductive diapause. Birth of viable offspring after arrival also could provide individuals of the opposite sex for reproduction (Nowack and Dausmann 2015).

## Risks of torpor use

In contrast to threats such as predation or storms (Turbill et al. 2011; Nowack et al. 2015), a sit-and-wait strategy during fires is only advantageous when animals are already hiding in a fire-safe refuge. Natural disasters can happen fast and unpredictably, giving the animal limited warnings, such as a drop in barometric pressure or the smell of smoke. Depending on the severity of the fire, smoke might be perceived relatively late by the individual, giving it only a short time to react. In this regard, torpor use might not only be beneficial, but also risky. If torpid animals cannot respond quickly to environmental threats, this would make them vulnerable and impose costs to the otherwise beneficial use of torpor. However, recent studies have shown that torpid animals can perceive olfactory and acoustic signals with *T*_b_s below 20 °C (Luo et al. 2014; Stawski et al. 2015b; Nowack et al. 2016b). Dense smoke as well as the smell of smoke (without the toxic substances) leads to an immediate termination of torpor and, therefore, could stimulate flight behaviour of torpid animals into more suitable shelters. Mobile animals are expected to either flee from the burning area or to search for shelter in underground burrows, caves, or tree hollows (reviewed in Engstrom 2010). Furthermore, several studies have shown that torpid animals are able to have coordinated movements with *T*_b_s as low as 14.8–17.9 °C, although locomotion at these low *T*_b_s is slow (Warnecke et al. 2008; Warnecke and Geiser 2010; Rojas et al. 2012; Nowack et al. 2016b).

## Longevity and torpor use

It has previously been suggested that predator avoidance could be one of the other functions of torpor (Geiser and Brigham 2012). This hypothesis has received further support from recent work on small mammals employing torpor after fires when predation pressure is likely to be increased, as described above. Along with allowing animals to avoid predation, it has also been proposed that torpor can increase longevity by enabling a “slow-paced” life history that is associated with increased survival rates during the hibernation season, slowed physiological aging, increased maximum longevity and long generation times (Lyman et al. 1981; Turbill et al. 2011, 2012, 2013; Ruf et al. 2012). Data on the relative length of telomeres (endcaps of DNA that shorten with every cell division) that are used as an indicator for physiological aging support this hypothesis (Turbill et al. 2012, 2013; Hoelzl et al. 2015, 2016). Meiotic cell divisions and erosion of telomeres via reactive oxygen-species are arrested or reduced at low *T*_b_s experienced during torpor (Kruman et al. 1988; Marcand et al. 2000) and reduced telomere shortening during the hibernation season has been reported in various studies (Turbill et al. 2012, 2013; Hoelzl et al. 2015, 2016). A correlation between torpor use and life spans in strepsirrhine primate species suggests that the extent of torpor expression is strongly linked with the life expectancy (Blanco and Godfrey 2013; Blanco and Zehr 2015). Similarly, torpor use and longevity were found to be correlated in Turkish hamsters (Lyman et al. 1981).

## Torpor and climate change

Climate change is expected to lead to an increase in occurrence and intensity of floods, storms, droughts, fires, and extreme temperatures (Christensen and Christensen 2003; CSIRO 2011). These events are expected to become the main causes of species extinction (McCain and King 2014). Wildfires are already increasingly being recognized as a major disturbance affecting many parts of the world, including Europe, Africa, America, Asia, and Australia. Progressing fragmentation, global warming, and human activities have led to frequent wildfires in ecosystems that were not classified as fire-prone (Moreira et al. 2001; Pechony and Shindell 2010). Furthermore, the occurrence of wildfires has increased in a number of regions over the past decades (e.g., Piñol et al. 1998). A gradually warming climate will also increase temperature extremes and, therefore, thermal stress on organisms, especially affecting desert species already coping with temperatures near their thermal limit. Heat waves already have had severe effects on many species and are especially challenging for species that do not shelter underground or in well-insulated nesting sites that offer protection from the heat, such as flying foxes (e.g., Welbergen et al. 2008).

Many seasonal hibernators, will likely face negative consequences as a result of the predicted increase in surface temperature. In the hazel dormouse (*Muscardinus avellanarius*), arousals from hibernation occurred more frequently during warmer years, causing substantial additional energy expenditure throughout the hibernation season (Pretzlaff and Dausmann 2012). Similarly, yellow-bellied marmots (*Marmota flaviventris*) emerged earlier from torpor than two decades earlier and this difference was attributed to the warmer spring *T*_a_ (Inouye et al. 2000). On the one hand, this increases the risk for especially small seasonal hibernators to deplete fat reserves before the end of hibernation. Humphries et al. (2002) calculated that an increase in *T*_a_ of 10 °C will increase the energy requirements of hibernating bats (*Myotis lucifugus*) threefold. On the other hand, climate change will also result in shorter winters, potentially associated with other problems, such as a reduction or a shift in the geographical range of species that are reliant on longer winters (Humphries et al. 2002).

Many of the functions of torpor would allow heterothermic animals to cope with the assumed impacts of climate change and, therefore, should give them an adaptive edge over homeothermic species with their constantly high energetic requirements. However, to be advantageous during unpredictable events, such as fires and storms, torpor use needs to be a flexible, opportunistic response, not the highly seasonal strategy employed by many species (Boyles et al. 2011). Particularly, multiday torpor use, i.e., seasonal hibernation, often requires preparation in the form of energy storage, either fat accumulation or food hoarding (Carey et al. 2003). Therefore, strictly seasonal hibernators will be less likely able to use torpor effectively if the challenges do not coincide with the hibernation season. However, there is ample evidence that the opportunistic use of short bouts of torpor throughout the year is not only expressed by daily heterotherms, but also is used by some hibernating species (reviewed in Levesque et al. 2016).

Phenotypic plasticity is higher in species that have evolved in variable habitats and this plasticity has been proposed to play an important role in vertebrate resilience in the light of climate change and habitat degradation (Canale and Henry 2010). Many heterothermic species are highly flexible in adjusting their energy requirements seasonally and regionally (e.g., Bozinovic et al. 2007; Boyles et al. 2013; Ruf and Geiser 2015) and often show pronounced differences in torpor use among populations of one species, individuals of one population, or even within individuals between different years (reviewed in Levesque et al. 2016). This flexibility is usually an acclimation or adaptation to habitats (e.g., habitat resources) or body condition. Heterotherms tend to undergo short-term torpor when body condition is poor, whereas multiday torpor is often expressed when body condition is good (Hallam and Mzilikazi 2011; Kobbe et al. 2011). Importantly, the phenotypic plasticity of energy expenditure afforded by the opportunistic use of torpor may be crucial in dealing with climate change and other anthropogenic disturbances (Nowack et al. 2015; Stawski et al. 2015a). The responses of heterothermic mammals to fires and storms are highly flexible and specific to individuals. While echidnas in an unburnt control habitat mostly used torpor synchronized in response to rainfall and *T*_a_ before the fire, echidnas adjusted *T*_b_ flexibly in response to varying resource distribution after the fire. Furthermore, brown antechinus (*A. stuartii*) displayed differences in torpor use between sexes (Stawski et al. 2016). This flexibility in behavioural and physiological traits suggests that heterothermic mammals are able to adjust readily to sudden environmental changes, and therefore, torpor use enables them to increase the likelihood to survive catastrophic events.

## Conclusion

Despite the risks associated with torpor, there is increasing evidence that the use of torpor is advantageous in many different settings and scenarios. While the removal of parasites is likely an additional benefit of cold *T*_b_, most functions, such as predator avoidance, development under reduced food sources, or disappearance from the surface during storms or fires could well be primary functions of torpor and serve a similar benefit as torpor use during winter. Heterothermic species are often able to cope with a wide *T*_a_ range, and perhaps, with the exception of strictly seasonal hibernators, many are likely to adjust better to a changing climate than homeotherms. Our review emphasises the many benefits of torpor use and suggests that especially the use of opportunistic torpor will give heterotherms an advantage over homeotherms during climate change.
